# The emergence of health inequalities in early adulthood: evidence on timing and mechanisms from a West of Scotland cohort

**DOI:** 10.1186/s12889-015-2674-5

**Published:** 2016-01-21

**Authors:** Helen Sweeting, Michael Green, Michaela Benzeval, Patrick West

**Affiliations:** 1 MRC/CSO Social & Public Health Sciences Unit, University of Glasgow, 200 Renfield Street, Glasgow, G2 3QB UK; 2 Institute for Social and Economic Research, University of Essex, Colchester, CO4 3SQ UK

## Abstract

**Background:**

Evidence is inconsistent as to whether or not there are health inequalities in adolescence according to socio-economic position (SEP) and whether or when they emerge in early adulthood. Despite the large health inequalities literature, few studies have simultaneously compared the relative importance of ‘health selection’ versus ‘social causation’ at this life-stage. This study followed a cohort through the youth-adult transition to: (1) determine whether, and if so, when, health inequalities became evident according to both class of origin and current SEP; (2) compare the importance of health selection and social causation mechanisms; and (3) investigate whether these phenomena vary by gender.

**Methods:**

Data are from a West-of-Scotland cohort, surveyed five times between age 15 (in 1987, *N*=1,515, response=85%) and 36. Self-reported physical and mental health were obtained at each survey. SEP was based on parental occupational class at 15, a combination of own education or occupational status at 18 and own occupational class (with an additional non-employment category) at older ages. In respect of when inequalities emerged, we used the relative index of inequality to examine associations between both parental and own current SEP and health at each age. In respect of mechanisms, path models, including SEP and health at each age, investigated both inter and intra-generational paths from SEP to health (‘causation’) and from health to SEP (‘selection’). Analyses were conducted separately for physical and mental health, and stratified by gender.

**Results:**

Associations between both physical and mental health and parental SEP were non-significant at every age. Inequalities according to own SEP emerged for physical health at 24 and for mental health at 30. There was no evidence of selection based on physical health, but some evidence of associations between mental health in early adulthood and later SEP (intra-generational selection). Paths indicated intra-generational (males) and inter-generational (females) social causation of physical health inequalities, and intra-generational (males and females) and inter-generational (females) social causation of mental health inequalities.

**Conclusions:**

The results suggest complex and reciprocal relationships between SEP and health and highlight adolescence and early adulthood as a sensitive period for this process, impacting on future life-chances and health.

**Electronic supplementary material:**

The online version of this article (doi:10.1186/s12889-015-2674-5) contains supplementary material, which is available to authorized users.

## Background

Although it is commonly assumed that social inequalities in health are a persistent feature of the life-course, this may vary across life stages and dimensions of health. Thus several [1–5], but not all studies [6–9] suggest ‘relative equality’ in adolescence [5, 10], with little or no differentiation on a range of health measures according to parental socioeconomic position (SEP), contrasting with inequalities found earlier in childhood and later in adulthood. In this paper, we address the questions of *when* and *how* [11] health inequalities emerge again in early adulthood. Our focus is thus the youth-adult transition, when individuals move from their parental SEP to their own (adult) position. To answer the ‘*when’* question, we examine associations between SEP (both parental and own current SEP) and health from mid-adolescence (age 15) to adulthood (age 36). To answer the ‘*how’* (mechanisms) question, we use structural equation modelling (SEM) including SEP and health at each age.

### When do health inequalities emerge?

Studies in the US and UK have found inequalities in respect of parental class emerging in people’s early 20s for measures of limiting longstanding illness, self-assessed health and depressive symptoms [12, 13]. However, some other studies suggest associations between parental SEP and health do not change in early adulthood, with inequalities either consistently present or consistently absent. For example, a Finnish cohort born in 1967 showed no differences according to social class at birth for self-assessed health or chronic illness at ages 16, 22 or 32 [14], whilst in the UK 1958 birth cohort, gradients according to social class at birth were present for school absence due to ill-health at age 16 [15] and for self-rated health, respiratory symptoms and malaise score at ages 22/23 and 32 [16]. Given these inconsistencies in whether or when inequalities emerge, it is clearly important to understand the mechanisms by which they can emerge [17]; answers as to how they emerge may inform expectations of whether and when they would emerge.

### How do health inequalities emerge?

Social inequalities in health may be due to the effects of ‘health selection’ (poorer health associated with downward, and good health with upward, social mobility) and/or ‘social causation’ (where occupational, educational and financial status influence health via material or cultural processes).

Many studies find that in late adolescence and early adulthood, health inequalities appear wider by reference to *own* class, education or labour market status than by *parental* SEP [2, 18–20]. Differences in chronic illness and self-reported health according to own education and social class emerged soon after age 20 and strengthened with age in UK and Finnish cross-sectional studies [12]. Strong associations between psychosomatic symptoms and own education at age 22 and own SEP at 32 were also seen in a Finnish cohort [21]. Although analysis of UK British Household Panel Survey data demonstrated no association between self-assessed health and own occupational class at age 20, social inequalities emerged by age 40 [22]. Comparison of the UK 1958 and 1970 birth cohorts also found inequalities in psychological distress according to own current social class which increased with age in the earlier cohort (ages 23, 33 and 42) but, and contrasting with all the above studies, decreased with age (26 and 30) in the later cohort [23]. The importance of a person’s own SEP suggests either health selection explanations, or that social causation effects are stronger from adult status than from childhood socioeconomic background.

A large research effort has focused on pathways between SEP and health from childhood/adolescence to early adulthood (*inter*-generational paths) and from earlier to later adulthood (*intra*-generational paths). For example, in respect of inter-generational health selection, evidence from the UK 1958 birth cohort has shown associations between several measures of poor childhood health and downward mobility from father’s to own adult occupational class and/or unemployment [15, 24–28]. Studies from Europe [29, 30] and the US [31, 32] highlight associations between poor health in childhood or adolescence and reduced educational attainment, temporary and non-employment. Intra-generational health selection is demonstrated by studies conducted in the UK [33], elsewhere in Europe [34–38] and the US [39] showing the importance of health for mobility in and out of employment, particularly among younger workers [39, 40]. Among the steadily employed, health may also affect promotional chances [41, 42].

A number of studies have investigated whether SEP in adulthood or early life is a more important determinant of adult health. A common finding is that early life and adult SEP are both independently associated with adult health [43]. For example, in the UK 1946 birth cohort, childhood social class, current SEP and circumstances, childhood illness and current physical activity were all associated, in mutually adjusted analyses, with poorer health at age 36 [44]. After parental class and health at ages 16 and 21 were controlled, adult social relations, labour market experiences, economic hardship and health behaviours all contributed to the socio-economic gradient in health at age 30 in a Swedish cohort [45], while a Danish study found low childhood and adult social class were both associated with health and social function in middle aged men [46]. A review found moderate support for the role of low socio-economic status in early life, and stronger support for the accumulated detrimental impact of low socio-economic status at multiple points across the life-course on cardiovascular risk factors [47].

Despite the vast literature focusing on the pathways between both childhood and adult health and SEP, few studies have used formal techniques such as SEM to compare the relative importance of time-lagged paths from health to SEP and from SEP to health simultaneously [48]. Among those which have, some have examined both inter- and intra-generational effects. Analyses of childhood, adolescent (age 16) and adult (ages 30–33) data from the British 1958 and 1970 birth cohorts demonstrated paths from adolescent distress/behaviour to own adult SEP (inter-generational selection), whilst paths representing social causation were evident at all life-stages [49]. Time-lagged longitudinal analyses of a Finnish cohort found a strong path from psychosomatic symptoms at age 16 to lower educational level at 22 (inter-generational selection), but no relationship between parental SEP at 16 and symptoms at 22 (inter-generational causation). There were also weaker paths, significant only for females, from symptoms at 22 to own SEP at age 32 and from own education at 22 to symptoms at 32 (intra-generational selection and causation) [21]. A US study, based on recalled child health ‘while you were growing up from birth to age 16’ found a complex net of paths, including: from parental SEP to child health, educational attainment and early occupational status; from child health to educational attainment, early and current occupational status, earnings, wealth and current self-rated health; and from educational attainment to early and current occupational status, earnings, wealth and current health [31]. These three studies, two from Europe focusing on psychological health and one from the US focusing on general health, suggest that both selection and causation processes are at work. However, two similar US studies, again examining inter- and intra-generational effects, found evidence of causation only [48, 50]. One of these, a study of SEP and mental illness, based on psychiatric hospitalisation and census data found no evidence that patients moved to lower SEP communities or experienced downward economic mobility after hospitalisation. Analyses limited to those who had experienced hospitalisation before age 18 also found no evidence of inter-generational downward mobility. In comparison, there was substantial evidence that SEP contributed to the development of mental illness [50]. The other US study, based on a cohort born in 1939 found no significant paths from health (self-rated; musculoskeletal; depression) to SEP at older ages nor from recalled childhood health to educational level. In contrast, evidence of social causation effects was ‘unambiguous’ [48].

SEM studies of adults (i.e. intra-generational effects), have all found strong evidence of causation, but less for selection. For example, there was strong evidence of an effect of SEP on changes in health, but only weak evidence of a selection effect of health (only among men, in respect of one health measure) on either changes in employment grade or financial deprivation among UK employees [51]. Despite this, selection effects were evident in some studies, in respect of: a factor representing physical illnesses, medication and self-rated health and SEP (education, income and occupation) among American adults followed for 20 years [52]; subjective well-being and subsequent career disruptions among Finnish managers [53]; and sickness allowance and later labour market disadvantage among both younger (35 years or less) and older adults in analyses of Finnish register data [54].

Overall, current evidence suggests health selection plays a relatively minor role in adult health inequalities compared with the inter- and intra-generational (social causation) effects of childhood and adulthood SEP [55–57]. However, further research is needed to understand the conditions, outcomes and populations for which these broad generalisations hold. In particular, there has been limited research which examines paths for men and women separately, despite the significantly different labour market experiences they have. In addition, much of this literature has employed broad measures of health, while it could be hypothesised that different kinds of health problems may have different impacts on SEP, and vice versa; indeed, it has been suggested that research comparing across different health measures would be a valuable way to increase understanding of SEP-health relationships [11].

### Differences by gender and health domain

It has been noted that gender is largely missing from work aiming to understand mechanisms linking SEP to health over the life-course [58]. Given gender differences in both biological disease processes [11] and experiences both within and outside the labour market [39], particularly in reasons for non-employment (mainly unemployment among males, but child/family care among females) [33], it is possible that health selection and social causation effects will differ between males and females. The SEM studies reviewed above have variously not included gender [49, 50], controlled for it [31, 53], found largely similar results for males and females [48, 52, 54] or small gender differences [21, 51]. Given these mixed results, it is important to investigate whether gender differences exist.

In addition, most studies in this area have represented both childhood/adolescent and adult health via measures of mental health, psychological well-being and/or psychosomatic symptoms [21, 25, 28–30, 32, 49, 50, 53]. Fewer have included physical health measures, although self-rated health and/or chronic conditions [26, 38] and sickness absences, allowances or hospital admissions [15, 34, 54] have been used. Many [24, 28–30, 32], but not all [48, 50] studies suggest that psychological distress has an impact on inter-generational mobility. However, chronic physical illness might also influence both inter- and intra-generational mobility; for example, increasing the likelihood of remaining in education rather than seeking employment [59], impacting negatively on educational achievement [60] and/or potentially leading to unemployment among those in manual rather than non-manual occupations [59, 61]. It might reasonably be expected that health selection and social causation effects would differ in respect of physical and mental health.

### Aims of this paper

In this paper we conduct a comprehensive analysis of social inequalities in health over the youth-adult transition and formally test the paths to their emergence in different health domains. This paper, therefore, provides the first systematic examination of whether inequalities in health emerge in early adulthood due to selection or causation. More specifically, the paper has three main aims:

1. To determine whether, and if so, when, inequalities in both physical and mental health become evident in respect of both class of origin and current SEP.
2. To use SEM to compare the importance of health selection and social causation mechanisms both inter-generationally and intra-generationally.
3. To investigate whether these phenomena vary by gender.

## Methods

### Participants

Data are from the 1970s cohort of the West of Scotland Twenty-07 Study [62], which consisted of 1,515 respondents resident in and around Glasgow, Scotland at baseline. Baseline interviews were carried out in 1987–88 when respondents were 15 years old, with follow-up interviews at ages 18, 24, 30 and 36 respectively, providing 20 years of follow-up. The baseline response rate was 85% and respondents have been shown to be representative of the general population of the sampled area [63]. Ethical approval for the overall study and baseline data collection was granted in 1986 by both the GP Sub-Committee of the Greater Glasgow Health Board Area Medical Committee and the Ethical Sub-Committee of the West of Scotland Medical Committee. Ethical approval for the fifth sweep of data collection was given by Tayside Committee on Medical Research Ethics A. At baseline, since the respondents were aged 15, informed written consent was sought from parents as well as respondents themselves. At the fifth sweep, informed written consent was given by the respondents. All authors are/were members of the Twenty-07 Study research team. Twenty-07 Study data are available to all bona fide researchers. Please see the data sharing policy - see http://2007study.sphsu.mrc.ac.uk/Data-Sharing-Policy.html for details.

### Measures

#### Physical health

Respondents were asked at each sweep whether they currently had any longstanding illness, disability or infirmity; to describe each condition and identify whether it limited their activities in any way. Conditions were coded according to the Royal College of General Practitioners classification scheme for morbidity data [64]. In order to ensure that this represented physical health only, limiting mental health conditions (codes 1000–1225) were excluded. The most prevalent physical health problems at age 15 were asthma, hayfever, migraines and skin conditions; at age 36 the physical conditions most frequently reported as limiting were asthma, migraines, hayfever, lower back pain and sciatica. A binary variable was constructed indicating whether, after excluding mental conditions, the respondent had any limiting, longstanding physical illness.

### Mental health

For mental health, validated symptom scales were preferred over self-reported mental health conditions since the latter were relatively rare (less than 3% at each survey); many young people had elevated symptom levels without actually reporting a mental health condition. There was no consistent mental health scale available for all five sweeps. The baseline measure of mental health was the 12-item General Health Questionnaire (GHQ-12) which was designed as a measure of state, focusing on inability to carry out normal functions and the emergence of distressing symptoms [65] and has been validated for use with adolescents [66]; scores of 2 or more were used to indicate poor mental health. The Hospital Anxiety and Depression Scale (HADS) [67, 68] was administered at each of the four follow-up sweeps. The HADS has two sub-scales; one for anxiety, and one for depression; for consistency with the GHQ-12, which provides an overall measure, a score of 8 or more on either sub-scale was used to indicate poor mental health [69].

### Socioeconomic position

It has been suggested that SEP conceptualisation and measurement “is among the more difficult and controversial subjects in social research” [70](p770), and that one of the properties of the “ideal” measure would be that it would permit analyses across the lifecourse. Unfortunately, there is no such measure. Socioeconomic position at age 15 was based on occupational class as reported by participants’ parents, coded according to the UK Registrar General’s 1980 classification [71] with values of 1 (professional/managerial) through to 6 (unskilled manual). The higher status occupation from working couples was used. Measurement of own SEP is particularly problematic for the categorisation of *young people post-school*, since many are in full-time tertiary education rather than working [72], and selection/causation studies including own occupational measures of SEP have varied in their methodological approach to this life-stage. Some have used educational attainment or trajectory as a proxy measure of future occupation [21]. This locates them at an appropriate position along their class trajectory at a life-stage when their own occupational class is often a poor indicator [72]. Others have combined education with occupational measures, generally placing those in tertiary education with those in professional or managerial positions [2, 73], consistent with the notion of tertiary education as associated with prestige [70]. Studies have also taken various approaches to those *outside the labour market*. This is an important conceptual distinction [51, 61]. A number of studies have restricted (some) analyses to men [24–26, 35] or to those in employment [51], while others have used most recent class for the non-employed [15, 49] or included non-employment as an additional occupational class [34, 35, 38]. The last approach has the twin advantages of not restricting analyses to certain groups or potentially biasing results by using non-concurrent social class and health measures.

Within our study, many respondents were still in full-time education at age 18 (25 % in Higher Education, 6 % Further Education, 1 % still at school), and this was considered indicative of a positive socioeconomic trajectory. Others were already in employment (46 % full-time, 3 % part-time, 6 % on a work-training scheme) and could be assigned an occupational class, while some were not in education or employment (13 %). The age 18 occupational class scores were therefore collapsed and combined with the respondents’ economic status as follows: 1=full-time education; 2=non-manual employment; 3=manual employment; 4=not in full-time education or employment. At each of the subsequent (adult) follow-ups, own current occupational class was used, coded according to the Registrar General’s 1980 classification (1 to 6) and respondents who were not in employment were assigned a score of 7 (indicating a more disadvantaged position).

### Statistical analysis

All analyses were conducted separately in respect of physical and mental health, and stratified by gender.

Firstly, in order to see *when* inequalities emerged, associations between SEP and health were examined with logistic regression at each age, comparing own, current SEP measures with the age 15 parental occupational class measure. As the SEP measures had different distributions we used the relative index of inequality (RII) [74] to compare them. This was done by ranking the respondents on each SEP measure and then assigning them scores based on their rank divided by the number of respondents. When used in logistic regression, the resulting odds ratios compare the odds of poor health for the least against the most affluent, adjusting for the different distributions of each measure.

Second, in order to investigate *how* inequalities emerged via selection and causation processes, path analyses were conducted in Mplus version 7 [75]. Missing data were assumed to be random, given the other variables in the model, and all respondents were included (*n*=737 for males and *n*=778 for females; 3 females were excluded in the analysis of mental health because of missing data on all variables). The path models included each measure of SEP and health regressed on all previous measurements of SEP and health (i.e. including all possible paths going forward in time). Apart from a correlation between baseline health and SEP, no cross sectional associations were included. Paths going forward in time are examined in preference to cross-sectional associations or overall trajectories of health and SEP, as the causal direction is less ambiguous when one variable clearly precedes another. All coefficients were standardised so the magnitude of selection and causation effects can be directly compared. Results are presented as path diagrams showing only paths with statistically significant coefficients. Paths from health to later SEP are viewed as representing selection effects, whilst paths from SEP to later health are viewed as representing causation effects. For both selection and causation effects, paths originating in adolescence (ages 15 and 18) and terminating in adulthood (ages 24+) are viewed as inter-generational, whilst paths originating and terminating in adulthood are viewed as intra-generational. Thus, rather than making arbitrary assumptions about the time-lag between health and SEP, the analysis allows for a range of selection and causation paths with differing time-lags. A Wald test was used to compare differences in model parameters between males and females, and a sensitivity analysis was conducted using the continuous scores for mental health (combining the anxiety and depression scores from the HADS into a total score).

## Results

Table 1 displays descriptive statistics for males and females at each survey sweep. SEP scores were most similar at age 15 (i.e. parental class). While the proportion of males working in non-manual occupations increased from around 40 % at age 24 to 70 % at 36, the proportion of females in non-manual occupations remained stable, at around two-thirds of the sample, throughout this early adulthood stage. The proportion of males not in employment also decreased between ages 24 and 36 while the proportion of females not in employment remained stable. These differences are reflected in the mean SEP scores which remained stable in females, but decreased in males from 3.99 (SD = 1.85) at age 24 to 3.20 (SD = 1.67) at age 36. Gender differences in reasons for non-employment were also evident. Of those shown as not in employment in Table 1, the proportions describing themselves as ‘caring for home or family’ were 0 % (males) and 47 % (females) at age 24, 2 % (m) and 51 % (f) at 30, and 3 % (m) and 53 % (f) at 36; the proportions of describing themselves as ‘unemployed’ were 53 % (m) and 0 % (f) at 24, 59 % (m) and 14 % (f) at 30, and 47 % (m) and 15 % (f) at 36. Thus, approximately half of the non-employed category for women was a result of home/family care, while half of the non-employed men were unemployed.

**Table 1:** Descriptive data for males and females on health and SEP measures at each age

	Age 15		Age 18		Age 24		Age 30		Age 36	
*N*										
Males	737		638		419		384		424	
Females	778		705		497		459		518	
Mean age (s.d.)										
Males	15.73	(0.33)	18.63	(0.34)	24.77	(0.98)	30.14	(1.30)	36.70	(0.43)
Females	15.76	(0.32)	18.65	(0.33)	24.86	(1.02)	30.20	(1.29)	36.74	(0.42)
Mean SEP score (s.d.)^a^										
Males	3.10	(1.25)	2.36	(1.06)	3.99	(1.85)	3.37	(1.75)	3.20	(1.67)
Females	3.18	(1.27)	2.09	(1.00)	3.68	(1.91)	3.52	(1.90)	3.56	(2.05)
*N* non-manual employment (%)										
Males	-	-	-	-	171	(41.6)	213	(55.8)	245	(59.3)
Females	-	-	-	-	322	(65.7)	312	(68.3)	317	(66.3)
*N* not in employment (%)^b^										
Males	-	-	85	(13.3)	77	(18.4)	44	(11.5)	38	(9.0)
Females	-	-	96	(13.6)	98	(19.7)	81	(17.6)	101	(19.5)
*N* LL physical illness (%)										
Males	64	(8.7)	64	(10.0)	57	(13.6)	69	(18.0)	81	(19.1)
Females	78	(10.0)	84	(11.9)	95	(19.1)	96	(20.9)	151	(29.2)
*N* poor mental health (%)^c^										
Males	135	(18.3)	215	(33.7)	129	(30.8)	125	(32.6)	142	(33.5)
Females	214	(27.5)	324	(46.0)	183	(36.8)	204	(44.4)	227	(43.8)

^a^Scores are as follows: Baseline is parent class (I=1, II=2, IIInm=3, IIIm=4, IV=5, V=6); 2^nd^ measurement is own educational and occupational status (full-time education=1, non-manual occupation=2, manual occupation=3, not in education or employment=4); 3^rd^-5^th^ measurements are own current class (I=1, II=2, IIInm=3, IIIm=4, IV=5, V=6, not in employment=7).

^b^Values for the second visit count only those not in education or employment.

^c^At baseline poor mental health is indicated by a GHQ-12 score ≥2, at all other visits by HADS sub-scale scores ≥8.

With respect to health, at all measurements females were more likely than males to experience poor mental health and report a limiting longstanding physical illness. It is important to note that although the two variables are conceptually distinct, there was a degree of co-morbidity. Table 2 shows the proportions with no morbidity, longstanding limiting physical illness only, poor mental health only or both at each age. Poor mental health was much more prevalent than a physical condition at every age; mental and physical co-morbidity increased from 4–5 % of the sample in adolescence to 13 % at age 36.

**Table 2:** No morbidity, longstanding limiting physical illness only, poor mental health only or both at each age^a^

	Age 15		Age 18		Age 24^b^		Age 30		Age 36	
	*N*	(%)	*N*	(%)	*N*	(%)	*N*	(%)	*N*	(%)
Neither	974	(69.6)	716	(54.0)	287	(43.9)	391	(49.1)	453	(49.0)
LL physical illness only	79	(5.6)	72	(5.4)	57	(8.7)	80	(10.0)	105	(11.4)
Poor mental health only	295	(21.1)	462	(34.9)	242	(37.0)	247	(31.0)	243	(26.3)
Both	51	(3.6)	75	(5.7)	68	(10.4)	79	(9.9)	124	(13.4)

^a^Analysis requires valid data on both longstanding limiting physical illness and poor mental health, thus numbers do not exactly tally with those in Table 1.

^b^Levels of missingness on poor mental health are high at age 24 because a portion of the sample only received a postal questionnaire that did not include the Hospital Anxiety and Depression Scale (HADS) instrument.

**Fig. 1:**
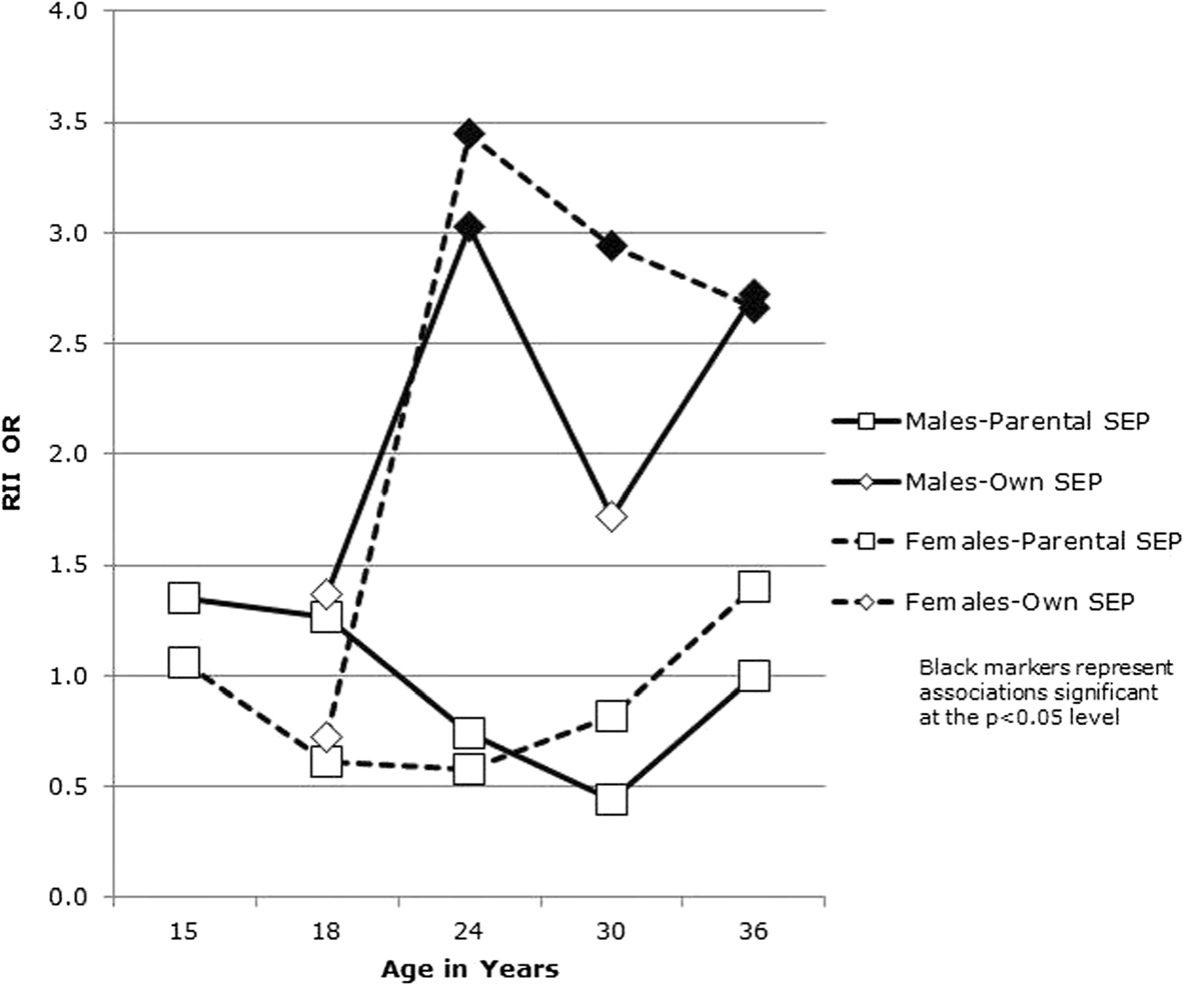
Poor physical health and SEP at each age

**Fig. 2:**
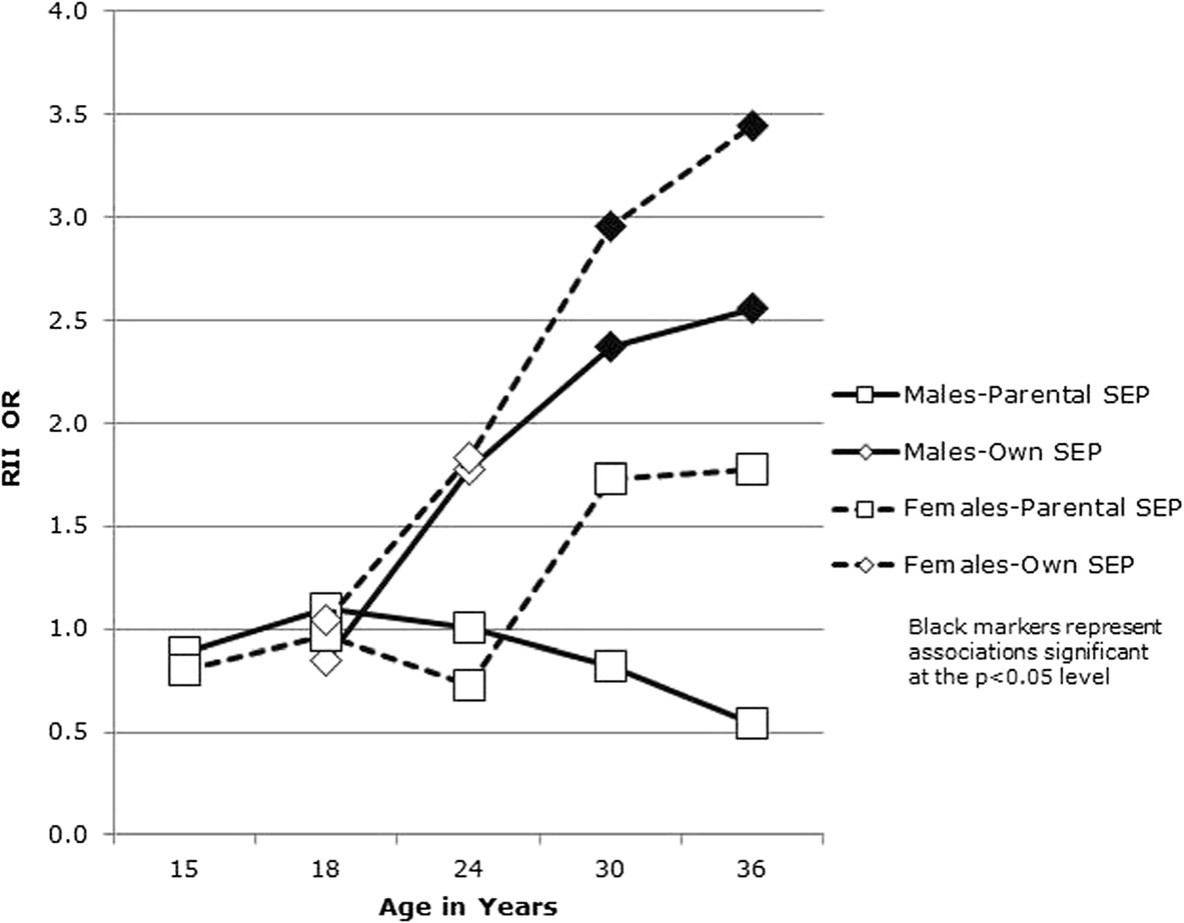
Poor mental health and SEP at each age

Focusing first on *when* inequalities emerged, Figs. 1 and 2 show the RII associations between SEP and health for males and females at each sweep. Associations for physical health are shown in Fig. 1 and those for mental health in Fig. 2. Associations between both physical and mental health measures and (age 15) parental occupational class were non-significant at every age. Additional file 1: Tables S1 and S2 (cross-tabulations of health at each age according to a range of age 15 SEP measures) show that this was not simply an artefact of the occupational class measure: a similar lack of association with health was evident in respect of parental income, parental education and deprivation of the home area. However, an association between own current SEP and physical health emerged for both males and females at age 24, and an association with mental health emerged for males and females at age 30. For males, the association between own current SEP and physical health dropped back out of significance at age 30 before re-emerging at age 36. Additional file 1: Tables S3 and S4 (cross-tabulations of health at each age according to own SEP at ages 18, 24, 30 and 36) show all associations between health and future SEP, and between SEP and future health. Although there were no linear associations between health and own current SEP at age 18, Additional file 1: Table S4 shows a U-shaped association in respect of mental health (highest levels of poor mental health among those not in education/work, followed by those in education and those in manual work, with those in non-manual work reporting the best mental health).

**Fig. 3:**
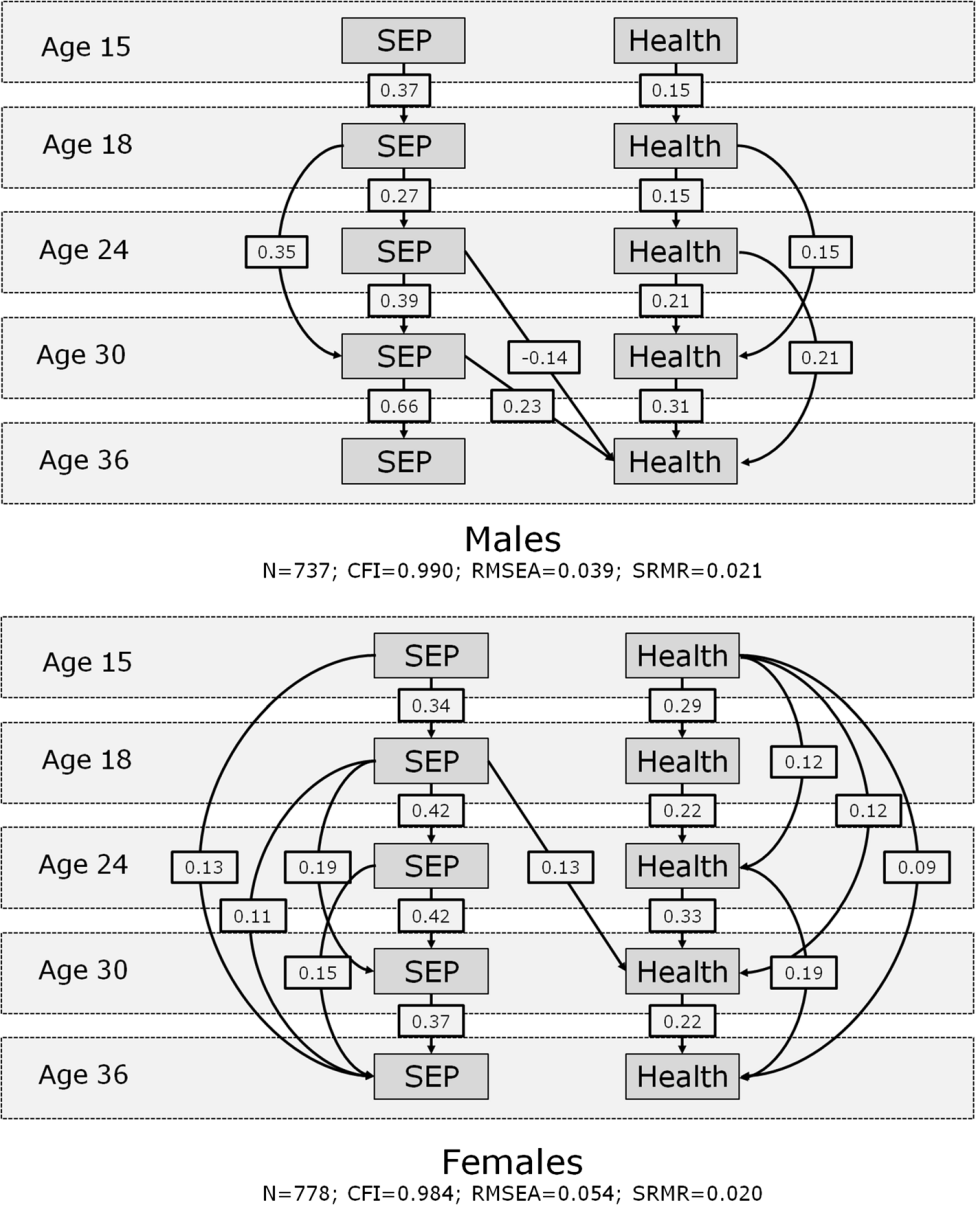
Path analysis of SEP and physical health from youth to adulthood

In respect of *how* inequalities emerged (causation/selection), Fig. 3 displays coefficients for the significant paths in the models relating to physical health for males and females (p<0.01 for gender difference). Both physical health and SEP were fairly stable over time, generally more stable for females than for males, and SEP was more stable than physical health. Effects indicating stability were present not just from the most recent prior measurement but also from earlier measurements, especially for females, suggesting a tendency to revert to earlier levels of physical health or SEP even after some deviation. Between ages 30–36, SEP (especially) and physical health were more stable among males than females.

None of the coefficients representing physical health selection effects on SEP were significant, but there was evidence of some causation effects. SEP at ages 24 and 30 was associated with physical health at age 36 for males and SEP at age 18 was associated with physical health at age 30 for females. Interestingly, for males, the two associations were in opposite directions: lower SEP at 24 was associated with *better* physical health at age 36 but lower SEP at age 30 was associated with *poorer* physical health at age 36, with the association in respect of SEP at age 24 being weaker than that in respect of SEP at age 30.

**Fig. 4:**
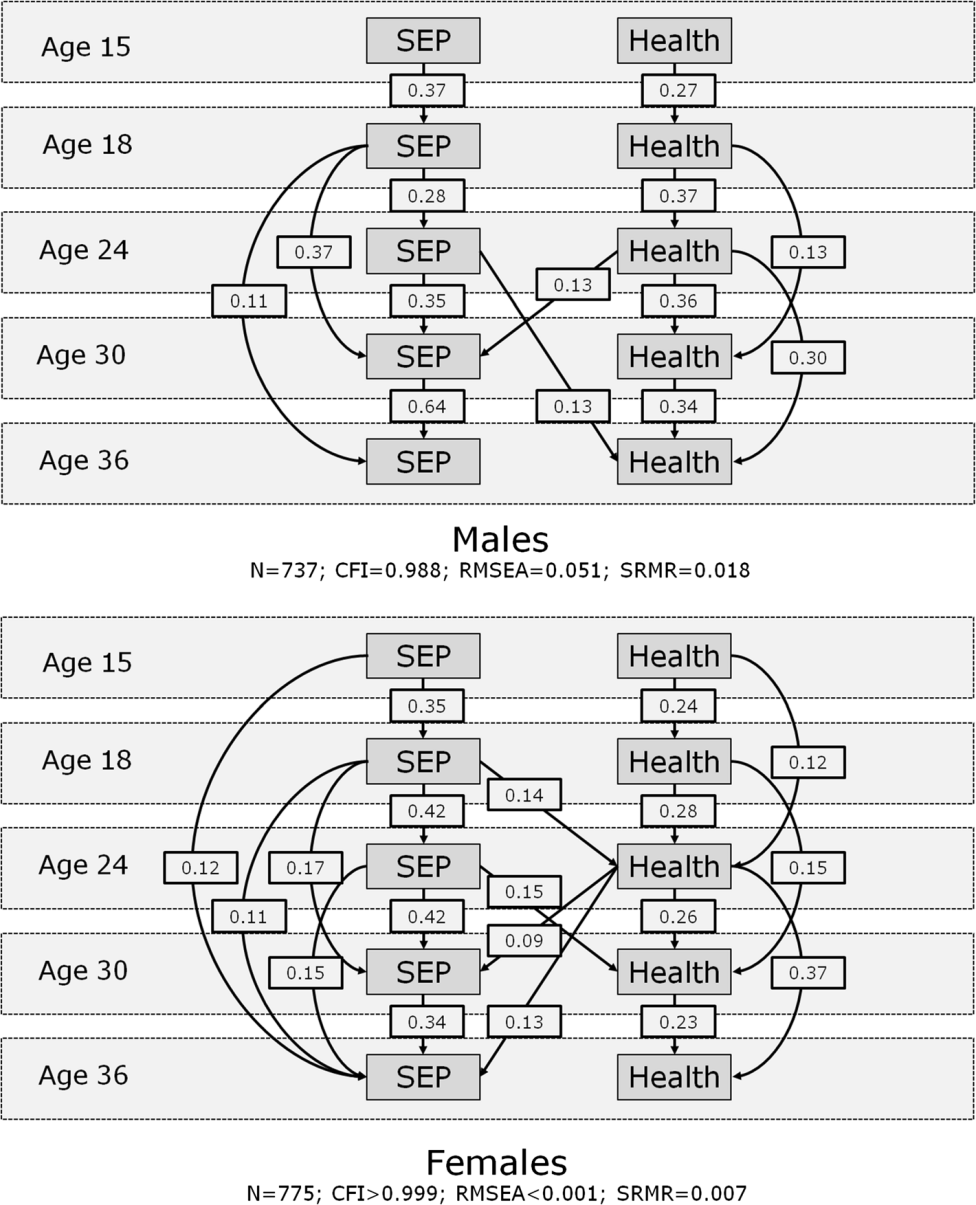
Path analysis of SEP and mental health from youth to adulthood

Figure 4 shows the significant paths from the male and female models relating to mental health and SEP (*p*=0.08 for gender difference). Mental health scores were more stable over time than physical health scores, and again there was evidence of stability, not just between consecutive measurements, but over the longer-term.

Both selection and causation effects were apparent for both males and females. Thus, for both males and females, there was a selection effect associating mental health at age 24 with SEP at the next measurement (age 30). Among females, age 24 mental health was also associated with age 36 SEP. This suggests intra-generational selection on the basis of mental health in early adulthood, particularly for females. Male SEP at age 24 exhibited an association with mental health at age 36, suggesting an intra-generational causation effect. For females on the other hand, there was evidence of inter-generational causation between ages 18 and 24 and intra-generational causation between ages 24 and 30.

Repeating the mental health models using continuous scores closely replicated the findings, except that an inter-generational selection effect linking poor mental health at age 18 to lower SEP at age 24 emerged for both males and females.

## Discussion

The first question addressed by this paper, based on a Scottish cohort, born in 1972 and followed from age 15 in 1987 to age 36 in 2007/8, was *whether*, and if so, *when* do health inequalities emerge in early adulthood? Results confirmed previous findings [5, 20, 76] of little or no variation in mid-late adolescent health in this cohort according to SEP. The present analysis also demonstrated no emergence of inequalities based on our measure of parental SEP (occupational class) measured when respondents were aged 15. Although some studies have found clear inequalities in health in adolescence [6–9], our findings are consistent with other analyses suggesting ‘relative equality’ [5, 10] in health in adolescents from the UK [2, 3], Australia [4], Canada [1] and those from a Finnish cohort, born around the same time (1967) which also found no associations between health and parental SEP post-adolescence [14]. However, in our analysis, inequalities in respect of own SEP emerged between ages 18 and 24 for physical health and between ages 24 and 30 for mental health. This is also consistent with most studies which have found greater evidence of health inequalities in respect of own, rather than parental SEP in early adulthood [2, 18–20] and that differences in health according to own SEP emerge in the early 20s and strengthen with increasing age over this period of the lifecourse [12, 21–23]. These analyses aimed to identify graded (linear) associations between SEP and health [77], and it is therefore worth noting the U-shaped cross-sectional relationship between own SEP at age 18 and mental health, with higher levels of poor mental health among those in education than those in work (but higher levels still among those in neither). A similar pattern was not evident in respect of age 18 SEP and mental health at older ages, suggesting a fairly transitory ‘student stress’ effect, also identified by others [78–80].

Our second question was *how* health inequalities emerge in early adulthood, and specifically the relative importance of health selection and social causation. We chose to stratify our analyses by gender, given the different labour market (and other) experiences of men and women [39]; much of the female SEP change in our study was a result of moving out of the labour market to look after the family, while that of men was associated with movement into non-manual occupations. Overall, more causation pathways were statistically significant than selection ones, and results differed for the two health measures and also between males and females. We did not find sufficient evidence of either selection or causation to explain the inequalities in physical health at age 24. This suggests that the association between physical health and own SEP at age 24 was independent of prior (ages 15 and 18) measures of health and SEP, and further study with shorter intervals between measurements around these ages is recommended.

We found no evidence of selection on the basis of physical health. However, among both males and females, poor mental health at age 24 was associated with more disadvantaged SEP at age 30 (and thus, better mental health at 24 was associated with more advantaged SEP); among females only, poorer mental health at 24 was also associated with more disadvantaged SEP at age 36. Gender differences in reasons for moving out of employment (i.e. looking after the family vs. unemployment) mean these findings may represent different health-related processes. Analyses using (more sensitive) continuous rather than binary mental health measures also found poor mental health at age 18 was associated with disadvantaged SEP at age 24, for both males and females. These results are in line with others suggesting an impact of psychological distress on (inter-generational) mobility [24, 28–30, 32]. The contrast in the findings between the binary and continuous mental health measures implies that some respondents with mild symptom levels at age 18 were doing particularly well in terms of SEP at age 24, contrasting with the tendency for those with more severe symptoms to do poorly in socioeconomic terms. This may represent anxieties associated with education [81], which could then lead to more positive socioeconomic outcomes.

Selection on the basis of mental but not physical health highlights the importance of recognising that different processes are likely to occur for different health measures. Young people moving from adolescence into early adulthood are in the process of establishing an adult identity, forming relationships, and negotiating transitions between social roles (e.g. from student to employee), and social environments (e.g. from secondary to tertiary education) [82, 83]. While common physical conditions at this stage of life, such as asthma, may impact on certain activities (e.g. athletics) or occupational choices [84], poor mental health may have a more significant influence on social, cognitive and psychological development [85]. Mechanisms, which may include academic/cognitive factors, peer relationships (perhaps related to stigma and/or stress associated with mental health), behavioural factors or aspirations/expectations [86–88] are likely to make it more difficult to manage these transitions successfully and emerge in an advantaged SEP [28]. A more general point is that low rates of poor physical health among young people mean it is unlikely to have a detectable effect on health inequalities in early adulthood, despite its potential importance for the lives of affected individuals [89]. Our own data accord with this: only around 10 % of the sample reported poor physical health at ages 15 or 18 (and only around 5 % as having poor physical health in the absence of poor mental health), and the most common physical condition in adolescence was asthma.

We found evidence of social causation for both health measures. Among males, there was evidence of intra-generational causation for mental health; disadvantaged SEP at age 24 was associated with poorer mental health at age 36. Disadvantaged SEP at 24 was also associated with *better* physical health at age 36, while disadvantaged SEP at age 30 was associated with *poorer* physical health at age 36, the latter association being slightly stronger. This suggests that among younger adult males, manual work (i.e. lower SEP) may have a positive effect on physical health, but that effect is lost if manual work continues. If the two effects are considered in combination, males with a disadvantaged SEP at both ages 24 and 30 will tend to have slightly poorer physical health overall.

Among females, there was evidence of both inter-generational causation (associations between disadvantaged SEP at age 18 and both poorer physical health at 30 and mental health at 24) and intra-generational causation (associations between disadvantaged SEP at age 24 and poorer mental health at 30). Among women, early childbirth is associated with disadvantaged, and later childbirth with advantaged SEP [90]. Disadvantaged SEP for women at age 18 may thus be particularly important because it could represent early parenthood as well as unemployment which, in turn, may significantly influence subsequent adult employment and SEP, and hence subsequent health. We found greater inequalities in psychological distress among females than males at ages 30 and 36, consistent with some other studies [91]. One reason might be that stresses in the lives of women resulting from combining low-paid, low-status status occupations or unemployment with family and household roles [92], exceed those of men.

In addition to gender differences, both selection and causation effects are likely to vary by age, time and place [11, 56]. In respect of age, there is evidence that health selection mechanisms are more likely at younger ages, around the stage of labour market entry, than in middle age [39, 40, 55, 93]. Our findings showed health selection mechanisms at this stage of life for mental but not physical health. In addition, educational attainment during this life-stage is both an SEP indicator and a determinant of other SEP indicators such as occupation and income [32] which, in turn, impact on future health. For both males and females in our analyses, the SEP indicator at age 18, which included educational status, was strongly associated with occupational success over the next 12–17 years of life, where there were patterns of intra-generational causation of health by SEP. Adolescence and young adulthood are therefore sensitive periods for future health and life-chances [21, 94, 95]. However, their importance seems strongly linked to the chains of risk initiated [57, 96–98], as we found young people’s own adult SEP to be more closely associated with adult health than parental SEP, and stronger evidence of intra-generational than of inter-generational causation and selection. Our results, along with those of others, highlight the complex mechanisms linking SEP and health [99] and the need to understand them as dynamic and interacting rather than mutually exclusive [11, 31].

In respect of time and place, our analyses tracked a UK, West of Scotland cohort from 1987 to 2007/8. The West of Scotland played a key part in the UK industrial revolution; by the mid-Twentieth century, half those in employment in the area worked in industry, after which manufacturing-based employment went into decline [100]. Over the 20-year period of the study, the contribution of manufacturing to Scotland’s economic output continued to decline with certain areas, including some from which the sample were drawn (e.g. North Lanarkshire) having difficulty replacing manufacturing-based employment, and therefore experiencing long-term unemployment. Despite this, total employment in Scotland grew over this period, almost entirely due to growth in public sector jobs [101]. Thus, while small numbers, particularly men, within our sample, may have experienced unemployment and socioeconomic deprivation, the sample overall is likely to have experienced improved material circumstances which might be expected to have resulted in overall improvements in health. However, this also means that those, again particularly men, who did experience downward mobility or move out of the labour force may have been more vulnerable in respect of individual circumstances or characteristics [102], which might include health. There is also evidence that health may have a greater effect on transitions in and out of employment than on mobility among the continuously employed [33, 42].

There is some evidence that societies where income is more equally distributed have better overall population health and fewer social problems [103], and it is possible that contextual differences might explain inconsistent results in studies of health inequalities. State-subsidised health and social care systems are less well-established in the US than Europe [104–106] which may be the reason that adolescent health inequalities are greater in the US [1]. Within the UK, where this study took place, the NHS has provided universal healthcare, free at the point of use, since 1948. The school system involves transition at age 11 from generally fairly small, local primary schools to much larger secondary schools with broader catchment areas and more scope for pupil mixing which may reduce adolescent health inequalities [107]. During the late 1980s and 1990s, when participants in our study made the transition from adolescence to early adulthood, successive governments responded to high youth unemployment levels with youth training schemes of variable quality [108]. In 1990, UK tertiary education divided into University/"Higher” and “Further Education”, (which provides most technical/vocational education and has traditionally been given much less attention and funding [109]) was free of fees. At that date moderate means-tested maintenance grants benefitting low income students were available, subsequently largely replaced by subsidised loans available to all [110]. However, despite the role of welfare states in mediating associations between SES and health, health inequalities are *not* consistently much less evident in countries with the most generous welfare regimes [11]. This ‘puzzle’ [104] means it is difficult to speculate on whether (or how) countries with different welfare regimes might vary in respect of mechanisms linking SES and health. We are unaware of any comprehensive reviews of evidence on selection and causation mechanisms in different welfare regimes; given suggestions that comparative research might increase understandings of processes linking SEP and health [11], this might be an area for further work.

Among the strengths of our study are the inclusion of five sweeps of data obtained around the post-school and early labour market periods, enabling a focus on both inter- and intra-generational mechanisms, and its approach to the measurement of own SEP, including non-employment at each age and educational status at age 18. We chose this (largely) occupationally-based SEP measure rather than education or income because education is generally complete in early adulthood (so selection on the basis of health would not be expected at older ages) and income was measured at the household level and so was complicated by whether an individual was in tertiary education and/or living in the parental home in late adolescence/early adulthood. It has been argued that different SEP measures are conceptually distinct and so may show different associations with health; there may even be subtle differences between parental wealth and current income in respect of their associations with adolescent physical health [111]. Our supplementary analyses (using parental income, education and deprivation of the home area) showed that the lack of association found between parental SEP and health at each age was not simply the result of having measured parental SEP via parental occupation, consistent with previous cross-sectional (age 15) analyses of this cohort [76]. We also included two health dimensions, physical and mental conditions, with only modest co-morbidity between them. It should be noted that our study was based on measures of health (albeit self-reported), rather than measures associated with health, such as childhood behavioural adjustment or health-related behaviours, which have also been shown to be related to future SEP [49, 112, 113]. Finally, our analyses were conducted separately for males and females, and identified gender differences in both patterns of movement between SEP categories and in pathways between SEP and health. However, our analyses also had some limitations. The Twenty-07 study began following these respondents when they were 15, so we cannot investigate associations between SEP and health at earlier ages, nor the impact of SEP and health throughout childhood on subsequent adult circumstances. There is only modest social mobility, both inter and intra generational, and, as noted above, movements in and out of the labour market have different meanings for men and women. We also acknowledge that whilst path models can establish temporal precedence, for example, between health and SEP, causal inference still depends on assumptions such as no unmeasured confounding and exchangeability [114]. We made the simplifying assumptions of treating some categorical variables as continuous, and restricting our analysis to linear associations, potentially missing any non-linear associations. However, additional analyses showed that almost every significant association was linear. The main exception (mental health according to current SEP at age 18, discussed above), was cross-sectional and our path models did not include cross-sectional associations. The mental health measures employed represent elevated symptom levels, rather than actual clinical diagnoses of anxiety or depression. In addition, we had to use a different mental health measure at the first (age 15) sweep from the other sweeps, though we do not feel this would have substantially affected our findings; both are psychiatric screening questionnaires with good discrimination between ‘cases’ and ‘non-cases’ [115].

## Conclusions

This study adds to the literature base in respect of health inequalities over the youth-adult transition and is therefore a step towards a more systematic understanding of associations between SEP and health and of how these may vary in different contexts. Overall, our results, like those of others in this area [31, 48, 49, 116] suggest complex and reciprocal relationships between SEP and health. What is needed now are studies which further unpack the ‘black box’ of factors linking SEP and health, since these may provide the possibility for interventions to improve both life-chances and health [17]. For example, aspects of the physical work environment may mediate social causation effects [17], while lack of support for young adults struggling with labour market entry or progression because of poor mental health may mediate health selection effects. Our results highlight the importance of taking a life-course perspective, recognising how social factors may accumulate and impact on life-chances as well as health, forming chains of risk [11, 31, 57, 96–98], and acknowledging adolescence [95] and early adulthood as very important stages in this process and therefore also as critical periods for intervention.

## Additional file

Additional file 1:
**Supplementary Tables 1-4 (health measures according to SEP at each age).**ᅟ
