# Supplementary material for: The emergence of health inequalities in early adulthood: evidence on timing and mechanisms from a West of Scotland cohort
Source: BMC Public Health. 2016 Jan 21;16:41. doi: 10.1186/s12889-015-2674-5 (PMC4721047; doi:10.1186/s12889-015-2674-5)
Supplement: Additional file 1: — Supplementary Tables 1-4 (health measures according to SEP at each age).ᅟ [file 12889_2015_2674_MOESM1_ESM.docx]

**Supplementary Table 1: Physical health (% longstanding limiting physical illness) at each age according to age 15 parental social class, income and education, and deprivation of home area – males and females**

|  |  |  |  |  |  |  |  |  |  |  |  |  |  |  |
| --- | --- | --- | --- | --- | --- | --- | --- | --- | --- | --- | --- | --- | --- | --- |
|  | **Age 15** | |  | **Age 18** | |  | **Age 24** | |  | **Age 30** | |  | **Age 36** | |
|  | Males | Females |  | Males | Females |  | Males | Females |  | Males | Females |  | Males | Females |
| **Parental social class** |  |  |  |  |  |  |  |  |  |  |  |  |  |  |
| Professional | 14.8 | 11.9 |  | 8.6 | 11.7 |  | 11.4 | 17.4 |  | 28.6 | 23.3 |  | 16.7 | 21.2 |
| Intermediate | 5.1 | 9.6 |  | 10.7 | 13.3 |  | 15.9 | 26.1 |  | 19.2 | 24.4 |  | 19.7 | 32.0 |
| Skilled non-manual | 10.3 | 10.5 |  | 8.3 | 13.6 |  | 14.3 | 12.4 |  | 18.8 | 17.1 |  | 21.0 | 24.2 |
| Skilled manual | 6.2 | 8.8 |  | 10.6 | 9.6 |  | 14.8 | 21.0 |  | 9.9 | 20.0 |  | 21.2 | 31.6 |
| Partly/unskilled | 13.9 | 11.8 |  | 12.2 | 9.2 |  | 9.3 | 14.7 |  | 18.6 | 22.4 |  | 14.9 | 32.5 |
| *(Overall X^2^ sig)* | *(0.021)* | *(0.909)* |  | *(0.882)* | *(0.667)* |  | *(0.793)* | *(0.053)* |  | *(0.167)* | *(0.684)* |  | *(0.881)* | *(0.349)* |
| *(Linear sig)* | *(0.487)* | *(0.932)* |  | *(0.595)* | *(0.252)* |  | *(0.598)* | *(0.240)* |  | *(0.086)* | *(0.614)* |  | *(0.959)* | *(0.349)* |
|  |  |  |  |  |  |  |  |  |  |  |  |  |  |  |
| **Parental income *** |  |  |  |  |  |  |  |  |  |  |  |  |  |  |
| Highest quintile | 8.5 | 10.8 |  | 11.9 | 10.3 |  | 13.4 | 16.3 |  | 22.7 | 18.1 |  | 23.0 | 26.4 |
| 4^th^ quintile | 10.9 | 9.7 |  | 8.7 | 13.8 |  | 17.8 | 18.9 |  | 18.2 | 20.2 |  | 15.5 | 26.9 |
| 3^rd^ quintile | 8.1 | 8.3 |  | 8.8 | 10.1 |  | 7.5 | 17.0 |  | 13.8 | 21.4 |  | 13.4 | 24.1 |
| 2^nd^ quintile | 10.3 | 11.6 |  | 12.9 | 14.3 |  | 18.1 | 27.5 |  | 17.5 | 27.9 |  | 24.4 | 40.2 |
| Lowest quintile | 5.0 | 10.6 |  | 8.8 | 10.2 |  | 10.0 | 18.7 |  | 15.7 | 16.7 |  | 18.6 | 31.6 |
| *(Overall X^2^ sig)* | *(0.426)* | *(0.908)* |  | *(0.706)* | *(0.695)* |  | *(0.243)* | *(0.410)* |  | *(0.666)* | *(0.557)* |  | *(0.348)* | *(0.129)* |
| *(Linear sig)* | *(0.321)* | *(0.853)* |  | *(0.825)* | *(0.970)* |  | *(0.598)* | *(0.338)* |  | *(0.250)* | *(0.701)* |  | *(0.912)* | *(0.120)* |
|  |  |  |  |  |  |  |  |  |  |  |  |  |  |  |
| **Parental education** |  |  |  |  |  |  |  |  |  |  |  |  |  |  |
| One/both post 16 | 10.8 | 12.5 |  | 9.1 | 14.2 |  | 15.2 | 20.9 |  | 22.8 | 21.5 |  | 20.9 | 24.0 |
| Neither parent post 16 | 7.8 | 9.0 |  | 10.6 | 10.4 |  | 12.9 | 17.3 |  | 14.5 | 20.4 |  | 18.4 | 31.9 |
| *(Overall X^2^ sig)* | *(0.174)* | *(0.137)* |  | *(0.552)* | *(0.141)* |  | *(0.506)* | *(0.317)* |  | *(0.040)* | *(0.795)* |  | *(0.515)* | *(0.054)* |
|  |  |  |  |  |  |  |  |  |  |  |  |  |  |  |
| **Area deprivation #** |  |  |  |  |  |  |  |  |  |  |  |  |  |  |
| Least deprived | 27.0 | 12.1 |  | 13.9 | 13.3 |  | 23.1 | 28.9 |  | 35.0 | 16.3 |  | 26.9 | 25.5 |
| 2 | 8.5 | 4.8 |  | 7.8 | 15.0 |  | 7.5 | 16.3 |  | 22.4 | 17.9 |  | 13.7 | 20.0 |
| 3 | 5.9 | 7.3 |  | 10.4 | 11.1 |  | 22.2 | 28.6 |  | 19.4 | 21.1 |  | 11.8 | 28.2 |
| 4 | 6.0 | 10.5 |  | 8.8 | 16.3 |  | 12.8 | 16.8 |  | 14.5 | 18.9 |  | 23.2 | 30.2 |
| 5 | 11.2 | 11.4 |  | 10.5 | 7.5 |  | 11.3 | 17.1 |  | 23.5 | 23.4 |  | 16.7 | 22.1 |
| 6 | 6.8 | 13.1 |  | 9.2 | 12.7 |  | 11.0 | 15.2 |  | 16.7 | 23.1 |  | 16.2 | 31.9 |
| Most deprived | 9.3 | 7.8 |  | 13.1 | 8.5 |  | 17.2 | 22.7 |  | 10.4 | 23.9 |  | 23.4 | 38.4 |
| *(Overall X^2^ sig)* | *(0.004)* | *(0.474)* |  | *(0.867)* | *(0.364)* |  | *(0.298)* | *(0.281)* |  | *(0.207)* | *(0.930)* |  | *(0.450)* | *(0.238)* |
| *(Linear sig)* | *(0.150)* | *(0.688)* |  | *(0.607)* | *(0.181)* |  | *(0.811)* | *(0.291)* |  | *(0.051)* | *(0.213)* |  | *(0.778)* | *(0.048)* |
|  |  |  |  |  |  |  |  |  |  |  |  |  |  |  |

* Income data obtained from parents via a banded income question. The mid-point of the chosen band was equivalised for household composition (McClements L. [1977] Equivalence scales for children. Journal of Public Economics 8: 191–210.) and then split into quintiles.

# Area deprivation, based on home postcodes coded by 1991 Census ‘Carstairs’ scores (Carstairs V, Morris R. [1991] Deprivation and health in Scotland. Aberdeen: Aberdeen University Press, 1991) and converted into standard area deprivation categories ranging from 1 (least) to 7 (most deprived).

**Supplementary Table 2: Poor mental health at each age according to age 15 parental social class, income and education, and deprivation of home area – males and females**

|  |  |  |  |  |  |  |  |  |  |  |  |  |  |  |
| --- | --- | --- | --- | --- | --- | --- | --- | --- | --- | --- | --- | --- | --- | --- |
|  | **Age 15** | |  | **Age 18** | |  | **Age 24** | |  | **Age 30** | |  | **Age 36** | |
|  | Males | Females |  | Males | Females |  | Males | Females |  | Males | Females |  | Males | Females |
| **Parental social class** |  |  |  |  |  |  |  |  |  |  |  |  |  |  |
| Professional | 11.7 | 40.7 |  | 31.0 | 37.3 |  | 27.3 | 48.3 |  | 28.6 | 34.9 |  | 30.2 | 43.1 |
| Intermediate | 23.8 | 31.9 |  | 37.2 | 49.5 |  | 49.0 | 50.0 |  | 40.0 | 43.4 |  | 44.8 | 40.8 |
| Skilled non-manual | 19.1 | 21.7 |  | 25.9 | 46.9 |  | 43.2 | 60.4 |  | 34.1 | 45.0 |  | 26.0 | 38.3 |
| Skilled manual | 17.1 | 31.7 |  | 36.6 | 44.5 |  | 35.0 | 52.6 |  | 27.5 | 50.6 |  | 35.4 | 53.1 |
| Partly/unskilled | 19.8 | 30.6 |  | 37.8 | 45.9 |  | 45.9 | 38.5 |  | 37.2 | 49.2 |  | 23.9 | 49.4 |
| *(Overall X^2^ sig)* | *(0.249)* | *(0.045)* |  | *(0.176)* | *(0.576)* |  | *(0.169)* | *(0.153)* |  | *(0.419)* | *(0.490)* |  | *(0.019)* | *(0.165)* |
| *(Linear sig)* | *(0.820)* | *(0.411)* |  | *(0.620)* | *(0.939)* |  | *(0.887)* | *(0.405)* |  | *(0.625)* | *(0.098)* |  | *(0.112)* | *(0.096)* |
|  |  |  |  |  |  |  |  |  |  |  |  |  |  |  |
| **Parental income *** |  |  |  |  |  |  |  |  |  |  |  |  |  |  |
| Highest quintile | 21.7 | 35.6 |  | 41.2 | 45.5 |  | 42.1 | 45.5 |  | 32.6 | 42.1 |  | 43.6 | 38.5 |
| 4^th^ quintile | 20.3 | 28.2 |  | 29.1 | 49.6 |  | 41.8 | 48.7 |  | 34.5 | 48.4 |  | 28.9 | 36.5 |
| 3^rd^ quintile | 15.3 | 26.7 |  | 29.4 | 45.9 |  | 40.7 | 51.4 |  | 39.1 | 44.6 |  | 28.8 | 45.3 |
| 2^nd^ quintile | 16.2 | 27.9 |  | 36.6 | 48.3 |  | 44.7 | 60.0 |  | 27.1 | 45.8 |  | 34.2 | 51.2 |
| Lowest quintile | 20.1 | 30.3 |  | 33.9 | 44.0 |  | 45.8 | 56.6 |  | 36.2 | 47.6 |  | 29.4 | 56.4 |
| *(Overall X^2^ sig)* | *(0.606)* | *(0.521)* |  | *(0.220)* | *(0.898)* |  | *(0.984)* | *(0.498)* |  | *(0.705)* | *(0.922)* |  | *(0.163)* | *(0.037)* |
| *(Linear sig)* | *(0.485)* | *(0.381)* |  | *(0.544)* | *(0.769)* |  | *(0.646)* | *(0.092)* |  | *(0.991)* | *(0.636)* |  | *(0.116)* | *(0.003)* |
|  |  |  |  |  |  |  |  |  |  |  |  |  |  |  |
| **Parental education** |  |  |  |  |  |  |  |  |  |  |  |  |  |  |
| One/both post 16 | 20.6 | 33.9 |  | 34.7 | 48.4 |  | 35.9 | 53.0 |  | 33.6 | 39.9 |  | 34.5 | 36.3 |
| Neither parent post 16 | 18.6 | 27.1 |  | 33.3 | 44.6 |  | 46.9 | 50.5 |  | 35.4 | 49.0 |  | 33.6 | 49.0 |
| *(Overall X^2^ sig)* | *(0.536)* | *(0.060)* |  | *(0.720)* | *(0.348)* |  | *(0.056)* | *(0.649)* |  | *(0.723)* | *(0.060)* |  | *(0.849)* | *(0.005)* |
|  |  |  |  |  |  |  |  |  |  |  |  |  |  |  |
| **Area deprivation #** |  |  |  |  |  |  |  |  |  |  |  |  |  |  |
| Least deprived | 35.1 | 28.6 |  | 40.0 | 53.3 |  | 45.8 | 51.1 |  | 40.0 | 51.2 |  | 30.8 | 34.0 |
| 2 | 25.0 | 44.1 |  | 29.9 | 50.0 |  | 35.9 | 53.1 |  | 31.9 | 39.5 |  | 34.0 | 36.4 |
| 3 | 16.7 | 31.5 |  | 33.3 | 35.2 |  | 32.4 | 38.1 |  | 31.2 | 37.8 |  | 28.1 | 43.6 |
| 4 | 18.4 | 28.5 |  | 32.7 | 42.4 |  | 42.4 | 50.7 |  | 30.4 | 38.5 |  | 39.8 | 41.0 |
| 5 | 16.7 | 21.0 |  | 36.4 | 46.7 |  | 42.5 | 56.9 |  | 42.0 | 60.0 |  | 33.3 | 48.1 |
| 6 | 17.8 | 28.9 |  | 37.2 | 48.1 |  | 42.9 | 54.1 |  | 38.7 | 44.8 |  | 30.0 | 45.6 |
| Most deprived | 16.9 | 33.8 |  | 32.1 | 48.1 |  | 51.4 | 57.9 |  | 34.0 | 50.8 |  | 32.8 | 54.1 |
| *(Overall X^2^ sig)* | *(0.176)* | *(0.090)* |  | *(0.901)* | *(0.498)* |  | *(0.765)* | *(0.606)* |  | *(0.804)* | *(0.140)* |  | *(0.816)* | *(0.282)* |
| *(Linear sig)* | *(0.028)* | *(0.564)* |  | *(0.861)* | *(0.961)* |  | *(0.281)* | *(0.288)* |  | *(0.599)* | *(0.397)* |  | *(0.798)* | *(0.012)* |
|  |  |  |  |  |  |  |  |  |  |  |  |  |  |  |

* Income data obtained from parents via a banded income question. The mid-point of the chosen band was equivalised for household composition (McClements L. [1977] Equivalence scales for children. Journal of Public Economics 8: 191–210.) and then split into quintiles.

# Area deprivation, based on home postcodes coded by 1991 Census ‘Carstairs’ scores (Carstairs V, Morris R. [1991] Deprivation and health in Scotland. Aberdeen: Aberdeen University Press, 1991) and converted into standard area deprivation categories ranging from 1 (least) to 7 (most deprived).

**Supplementary Table 3: Physical health (% longstanding limiting physical illness) at each age according to own SEP at ages 18, 24, 30 and 36 – males and females**

|  |  |  |  |  |  |  |  |  |  |  |  |  |  |  |
| --- | --- | --- | --- | --- | --- | --- | --- | --- | --- | --- | --- | --- | --- | --- |
|  | **Age 15** | |  | **Age 18** | |  | **Age 24** | |  | **Age 30** | |  | **Age 36** | |
|  | Males | Females |  | Males | Females |  | Males | Females |  | Males | Females |  | Males | Females |
| **Own age 18 SEP** |  |  |  |  |  |  |  |  |  |  |  |  |  |  |
| Full-time education | 6.7 | 8.4 |  | 10.9 | 12.7 |  | 12.8 | 20.6 |  | 17.9 | 14.4 |  | 21.5 | 23.6 |
| Non-manual work | 8.9 | 10.0 |  | 7.0 | 12.7 |  | 15.5 | 16.9 |  | 21.1 | 21.2 |  | 19.4 | 26.4 |
| Manual work | 7.2 | 10.9 |  | 9.2 | 9.9 |  | 10.8 | 19.7 |  | 16.2 | 32.8 |  | 14.7 | 34.9 |
| Not f/t education/work | 15.9 | 12.5 |  | 15.3 | 10.4 |  | 19.6 | 25.0 |  | 14.3 | 28.3 |  | 23.7 | 47.1 |
| *(Overall X^2^ sig)* | *(0.070)* | *(0.714)* |  | *(0.270)* | *(0.829)* |  | *(0.435)* | *(0.574)* |  | *(0.771)* | *(0.018)* |  | *(0.402)* | *(0.006)* |
| *(Linear sig)* | *(0.094)* | *(0.247)* |  | *(0.549)* | *(0.432)* |  | *(0.714)* | *(0.628)* |  | *(0.518)* | *(0.004)* |  | *(0.444)* | *(0.001)* |
|  |  |  |  |  |  |  |  |  |  |  |  |  |  |  |
| **Own age 24 SEP** |  |  |  |  |  |  |  |  |  |  |  |  |  |  |
| Professional | 6.5 | 11.1 |  | 9.7 | 10.7 |  | 6.7 | 10.7 |  | 12.0 | 12.0 |  | 22.7 | 16.7 |
| Intermediate | 7.5 | 8.3 |  | 16.4 | 11.9 |  | 13.4 | 15.5 |  | 19.1 | 20.0 |  | 21.8 | 22.4 |
| Skilled non-manual | 8.2 | 6.9 |  | 4.2 | 10.1 |  | 11.0 | 18.6 |  | 18.6 | 18.1 |  | 11.9 | 25.9 |
| Skilled manual | 9.3 | 4.3 |  | 11.2 | 0.0 |  | 11.1 | 17.4 |  | 15.2 | 27.8 |  | 11.4 | 22.2 |
| Partly/unskilled | 11.1 | 12.8 |  | 7.3 | 13.0 |  | 16.4 | 13.3 |  | 18.9 | 23.3 |  | 18.9 | 22.6 |
| Not employed | 7.8 | 14.3 |  | 6.8 | 16.8 |  | 22.4 | 33.3 |  | 24.1 | 32.8 |  | 18.5 | 38.6 |
| *(Overall X^2^ sig)* | *(0.973)* | *(0.348)* |  | *(0.184)* | *(0.303)* |  | *(0.195)* | *(0.009)* |  | *(0.773)* | *(0.174)* |  | *(0.478)* | *(0.172)* |
| *(Linear sig)* | *(0.751)* | *(0.081)* |  | *(0.225)* | *(0.169)* |  | *(0.019)* | *(0.001)* |  | *(0.257)* | *(0.011)* |  | *(0.790)* | *(0.014)* |
|  |  |  |  |  |  |  |  |  |  |  |  |  |  |  |
| **Own age 30 SEP** |  |  |  |  |  |  |  |  |  |  |  |  |  |  |
| Professional | 10.0 | 6.9 |  | 5.1 | 10.0 |  | 9.7 | 11.1 |  | 15.0 | 20.0 |  | 21.2 | 17.2 |
| Intermediate | 6.0 | 11.8 |  | 7.1 | 13.5 |  | 17.4 | 16.2 |  | 16.5 | 16.9 |  | 12.3 | 23.3 |
| Skilled non-manual | 10.7 | 4.8 |  | 5.5 | 6.4 |  | 8.2 | 17.1 |  | 16.4 | 16.1 |  | 16.3 | 24.2 |
| Skilled manual | 8.5 | 4.8 |  | 10.1 | 4.8 |  | 11.4 | 17.6 |  | 19.1 | 19.0 |  | 8.1 | 31.6 |
| Partly/unskilled | 13.3 | 18.6 |  | 12.9 | 15.0 |  | 8.0 | 28.1 |  | 12.9 | 37.2 |  | 19.2 | 34.4 |
| Not employed | 9.3 | 9.9 |  | 7.1 | 12.7 |  | 19.4 | 27.4 |  | 27.3 | 29.6 |  | 34.5 | 33.8 |
| *(Overall X^2^ sig)* | *(0.807)* | *(0.086)* |  | *(0.749)* | *(0.326)* |  | *(0.452)* | *(0.258)* |  | *(0.591)* | *(0.016)* |  | *(0.023)* | *(0.370)* |
| *(Linear sig)* | *(0.542)* | *(0.541)* |  | *(0.439)* | *(0.666)* |  | *(0.832)* | *(0.017)* |  | *(0.157)* | *(0.004)* |  | *(0.087)* | *(0.031)* |
|  |  |  |  |  |  |  |  |  |  |  |  |  |  |  |
| **Own age 36 SEP** |  |  |  |  |  |  |  |  |  |  |  |  |  |  |
| Professional | 12.8 | 13.3 |  | 10.5 | 15.6 |  | 10.3 | 20.0 |  | 17.1 | 25.9 |  | 20.5 | 18.8 |
| Intermediate | 7.6 | 9.3 |  | 9.2 | 15.5 |  | 13.2 | 16.7 |  | 19.7 | 20.4 |  | 14.6 | 27.3 |
| Skilled non-manual | 10.4 | 6.9 |  | 4.7 | 5.3 |  | 8.1 | 16.7 |  | 14.3 | 13.4 |  | 16.7 | 29.4 |
| Skilled manual | 6.3 | 15.4 |  | 8.0 | 0.0 |  | 11.9 | 11.1 |  | 13.8 | 0.0 |  | 14.7 | 23.1 |
| Partly/unskilled | 8.8 | 8.5 |  | 12.1 | 13.0 |  | 8.7 | 19.4 |  | 25.0 | 28.2 |  | 26.5 | 21.3 |
| Not employed | 10.8 | 13.9 |  | 15.2 | 17.6 |  | 29.2 | 25.8 |  | 27.3 | 31.8 |  | 39.5 | 44.6 |
| *(Overall X^2^ sig)* | *(0.826)* | *(0.594)* |  | *(0.704)* | *(0.086)* |  | *(0.222)* | *(0.681)* |  | *(0.654)* | *(0.048)* |  | *(0.010)* | *(0.012)* |
| *(Linear sig)* | *(0.967)* | *(0.299)* |  | *(0.414)* | *(0.547)* |  | *(0.132)* | *(0.156)* |  | *(0.504)* | *(0.057)* |  | *(0.004)* | *(0.003)* |
|  |  |  |  |  |  |  |  |  |  |  |  |  |  |  |

**Supplementary Table 4: Poor mental health at each age according to own SEP at ages 18, 24, 30 and 36 – males and females**

|  |  |  |  |  |  |  |  |  |  |  |  |  |  |  |
| --- | --- | --- | --- | --- | --- | --- | --- | --- | --- | --- | --- | --- | --- | --- |
|  | **Age 15** | |  | **Age 18** | |  | **Age 24** | |  | **Age 30** | |  | **Age 36** | |
|  | Males | Females |  | Males | Females |  | Males | Females |  | Males | Females |  | Males | Females |
| **Own age 18 SEP** |  |  |  |  |  |  |  |  |  |  |  |  |  |  |
| Full-time education | 23.0 | 34.1 |  | 39.9 | 51.1 |  | 37.3 | 49.2 |  | 33.6 | 43.8 |  | 36.0 | 38.3 |
| Non-manual work | 24.2 | 25.4 |  | 26.7 | 38.9 |  | 50.0 | 49.3 |  | 38.8 | 44.5 |  | 33.9 | 45.7 |
| Manual work | 14.8 | 23.1 |  | 29.3 | 46.0 |  | 40.2 | 59.5 |  | 30.1 | 56.4 |  | 32.9 | 53.2 |
| Not f/t education/work | 16.5 | 42.9 |  | 43.4 | 57.9 |  | 46.9 | 63.6 |  | 42.1 | 43.2 |  | 40.5 | 52.0 |
| *(Overall X^2^ sig)* | *(0.076)* | *(0.004)* |  | *(0.011)* | *(0.004)* |  | *(0.420)* | *(0.314)* |  | *(0.453)* | *(0.397)* |  | *(0.826)* | *(0.117)* |
| *(Linear sig)* | *(0.031)* | *(0.593)* |  | *(0.547)* | *(0.418)* |  | *(0.488)* | *(0.097)* |  | *(0.865)* | *(0.504)* |  | *(0.965)* | *(0.022)* |
|  |  |  |  |  |  |  |  |  |  |  |  |  |  |  |
| **Own age 24 SEP** |  |  |  |  |  |  |  |  |  |  |  |  |  |  |
| Professional | 13.3 | 19.2 |  | 25.8 | 44.4 |  | 35.0 | 55.0 |  | 32.0 | 40.0 |  | 13.6 | 36.0 |
| Intermediate | 25.4 | 37.6 |  | 32.8 | 46.2 |  | 32.6 | 42.9 |  | 27.7 | 40.4 |  | 34.5 | 41.8 |
| Skilled non-manual | 20.5 | 25.1 |  | 30.6 | 43.2 |  | 45.6 | 51.5 |  | 35.7 | 40.3 |  | 29.3 | 42.4 |
| Skilled manual | 15.0 | 23.8 |  | 29.2 | 65.2 |  | 40.0 | 64.7 |  | 28.9 | 76.5 |  | 32.9 | 66.7 |
| Partly/unskilled | 16.4 | 38.1 |  | 34.5 | 50.0 |  | 44.7 | 63.3 |  | 37.1 | 50.0 |  | 37.8 | 35.5 |
| Not employed | 24.0 | 32.3 |  | 39.2 | 50.5 |  | 47.5 | 54.7 |  | 32.7 | 56.2 |  | 43.4 | 51.5 |
| *(Overall X^2^ sig)* | *(0.434)* | *(0.139)* |  | *(0.717)* | *(0.443)* |  | *(0.645)* | *(0.356)* |  | *(0.910)* | *(0.028)* |  | *(0.218)* | *(0.208)* |
| *(Linear sig)* | *(0.714)* | *(0.630)* |  | *(0.177)* | *(0.286)* |  | *(0.164)* | *(0.214)* |  | *(0.738)* | *(0.016)* |  | *(0.032)* | *(0.164)* |
|  |  |  |  |  |  |  |  |  |  |  |  |  |  |  |
| **Own age 30 SEP** |  |  |  |  |  |  |  |  |  |  |  |  |  |  |
| Professional | 18.4 | 27.6 |  | 35.9 | 31.0 |  | 28.6 | 30.0 |  | 35.0 | 31.0 |  | 26.5 | 25.0 |
| Intermediate | 20.5 | 33.1 |  | 30.7 | 46.2 |  | 36.9 | 51.9 |  | 25.9 | 40.2 |  | 32.7 | 43.3 |
| Skilled non-manual | 18.5 | 25.2 |  | 27.3 | 51.8 |  | 30.8 | 52.7 |  | 43.6 | 44.7 |  | 25.6 | 44.7 |
| Skilled manual | 10.3 | 25.0 |  | 25.0 | 55.0 |  | 42.6 | 53.8 |  | 25.0 | 57.1 |  | 28.4 | 35.0 |
| Partly/unskilled | 6.7 | 15.4 |  | 48.4 | 37.5 |  | 52.4 | 50.0 |  | 41.9 | 48.8 |  | 42.3 | 41.9 |
| Not employed | 26.2 | 30.7 |  | 35.7 | 53.2 |  | 40.0 | 63.3 |  | 59.5 | 59.0 |  | 39.3 | 52.9 |
| *(Overall X^2^ sig)* | *(0.125)* | *(0.334)* |  | *(0.220)* | *(0.198)* |  | *(0.536)* | *(0.268)* |  | *(0.001)* | *(0.051)* |  | *(0.594)* | *(0.217)* |
| *(Linear sig)* | *(0.881)* | *(0.627)* |  | *(0.545)* | *(0.271)* |  | *(0.208)* | *(0.058)* |  | *(0.006)* | *(0.002)* |  | *(0.293)* | *(0.070)* |
|  |  |  |  |  |  |  |  |  |  |  |  |  |  |  |
| **Own age 36 SEP** |  |  |  |  |  |  |  |  |  |  |  |  |  |  |
| Professional | 21.1 | 22.6 |  | 34.2 | 41.9 |  | 30.4 | 40.0 |  | 30.3 | 40.7 |  | 23.1 | 21.9 |
| Intermediate | 20.6 | 27.1 |  | 34.6 | 41.3 |  | 36.4 | 40.2 |  | 32.5 | 37.7 |  | 32.3 | 42.3 |
| Skilled non-manual | 19.1 | 26.7 |  | 27.9 | 47.3 |  | 28.6 | 52.8 |  | 47.1 | 37.0 |  | 36.2 | 39.6 |
| Skilled manual | 11.1 | 23.1 |  | 27.6 | 69.2 |  | 47.1 | 80.0 |  | 21.9 | 77.8 |  | 20.0 | 61.5 |
| Partly/unskilled | 8.6 | 27.9 |  | 28.1 | 60.9 |  | 47.4 | 52.6 |  | 45.5 | 51.4 |  | 42.9 | 58.7 |
| Not employed | 32.4 | 34.1 |  | 51.5 | 56.0 |  | 52.9 | 65.3 |  | 45.0 | 59.0 |  | 67.6 | 57.1 |
| *(Overall X^2^ sig)* | *(0.051)* | *(0.790)* |  | *(0.199)* | *(0.046)* |  | *(0.352)* | *(0.045)* |  | *(0.091)* | *(0.013)* |  | *(0.000)* | *(0.002)* |
| *(Linear sig)* | *(0.888)* | *(0.175)* |  | *(0.389)* | *(0.006)* |  | *(0.051)* | *(0.003)* |  | *(0.417)* | *(0.002)* |  | *(0.001)* | *(0.000)* |
|  |  |  |  |  |  |  |  |  |  |  |  |  |  |  |
